# Fabrication of Fe-Based Diamond Composites by Pressureless Infiltration

**DOI:** 10.3390/ma9121006

**Published:** 2016-12-12

**Authors:** Meng Li, Youhong Sun, Qingnan Meng, Haidong Wu, Ke Gao, Baochang Liu

**Affiliations:** 1 College of Construction Engineering, Jilin University, Changchun 130000, China; mli14@mails.jlu.edu.cn (M.L.); syh@jlu.edu.cn (Y.S.); qingnanmeng@jlu.edu.cn (Q.M.); wuhd12@mails.jlu.edu.cn (H.W.); 2 Key Laboratory of Drilling and Exploitation Technology in Complex Conditions of Minsitry of Land and Resources, Changchun 130000, China

**Keywords:** impregnated diamond composites, pressureless infiltration, wear resistance, boron

## Abstract

A metal-based matrix is usually used for the fabrication of diamond bits in order to achieve favorable properties and easy processing. In the effort to reduce the cost and to attain the desired bit properties, researchers have brought more attention to diamond composites. In this paper, Fe-based impregnated diamond composites for drill bits were fabricated by using a pressureless infiltration sintering method at 970 °C for 5 min. In addition, boron was introduced into Fe-based diamond composites. The influence of boron on the density, hardness, bending strength, grinding ratio, and microstructure was investigated. An Fe-based diamond composite with 1 wt % B has an optimal overall performance, the grinding ratio especially improving by 80%. After comparing with tungsten carbide (WC)-based diamond composites with and without 1 wt % B, results showed that the Fe-based diamond composite with 1 wt % B exhibits higher bending strength and wear resistance, being satisfactory to bit needs.

## 1. Introduction

Impregnated diamond composite bits are widely used in drilling exploration. The matrix composition and crown shape of the diamond bit are the two key factors determining the bit quality [1,2,3,4,5], and both are related to the sintering process. For impregnated diamond bits, diamond grits are distributed into the matrix as abrasive particles; the matrix should bond the diamond grits tightly and be worn in a proper rate for exposed fresh diamond grits. Impregnated diamond bits are usually fabricated by the hot press sintering method [6,7,8,9]. Common weaknesses of hot press sintering mainly includes two problems. On one hand, the conventional metal-based matrix is complex, containing tungsten carbide (WC), Fe, Co, Cu, Mn, Zn, Sn, etc., both different power and size making it difficult to mix and causing the segregation of components. On the other hand, the hot press method has a restricting effect on the bits’ special shape. However, the pressureless infiltration sintering method can be considered in the fabrication of bits [10,11,12]. WC and Ni are widely used as base bit matrix for pressureless infiltration sintering, and Cu alloy is used as a binding matrix by capillary force. Fewer components can avoid component segregation, and pressureless infiltration can be conducive to molding. For the fabrication of impregnated diamond bits, WC is always used as a framework material for its high hardness and high wear-resistance. In recent years, Fe-based matrices have been widely used in diamond tool manufacturing for its good mechanical properties and lower cost. Fe plays a dual role in diamond composites. Fe has a good wettability to many metals and diamond, improving the matrix alloying and the ability of matrix to hold diamond. However, Fe would heavily erode the diamond grits’ surface under high temperature, greatly reducing the cutting ability of diamond. Since Fe-based impregnated diamond bits show a very good development prospect, many researchers have carried out studies to prevent or reduce the eroding phenomenon through additions, diamond coating, and sintering parameters optimization [13,14,15,16,17,18]. Boron (B) or Boride have a good effect on the diamond composites, especially in aspects of wetting ability, wear resistance, thermal ability and chemical stability [19,20,21,22].

In this paper, Fe-based diamond composites with B were fabricated by pressureless infiltration, and were compared with the properties of WC-based diamond composites. The main aim of this paper is to investigate the effect of B on the microstructure and properties of Fe-based diamond composites. Relative density, hardness, bending strength, and wear resistance were tested in order to estimate the properties of diamond composites and to investigate the microstructures of diamond composites.

## 2. Materials and Methods

Fe-based diamond composites were prepared by pressureless infiltration sintering in order to compare properties. Commercial powders, including Fe (99.8% purity, average particle size 150 μm) and 10% Ni (99.8% purity, average particle size 100 μm) were used as the base components of the diamond composites. Cu alloy (50 wt %; BC-3, Cu-Ni16%-Mn25%-Zn10%-Si0.2%) was used as the binder. Moreover, 20 vol % diamond grits (MBD-6, average particle size 325–380 μm) were employed as abrasive particles. Boron (B) powder (purity >99.99 wt %, average particle size 38 μm) was used as the additive.

**Figure 1:**
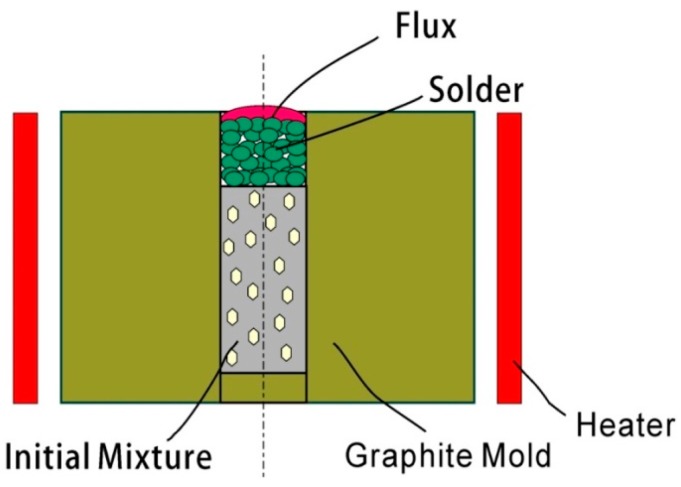
Schematic diagram of pressureless infiltration. Sample size: φ8 mm × 32 mm.

Figure 1 demonstrates the principle of pressureless infiltration. The diamond composite samples were fabricated by pressureless infiltration sintering according to the following procedure. Firstly, Fe powder and Ni powder were blended in a three-dimensional mixer with ball-miller for 8 h at 120 rpm in order to attain the initial Fe-based matrix. The different contents of B powder (Table 1) were mixed into the initial matrix mixtures using a ball-miller for 2 h at the same speed. Diamond grits were added into the matrix mixture using alcohol and ultrasonic vibration to prepare the diamond composite mixture. Then, the diamond composite mixture was put into graphite dye for 30 min to remove the alcohol and was then covered with Cu alloy and brazing flux. Finally, graphite dye was sintered at 970 °C for 5 min in a mid-frequency induction furnace, then cooled down to room temperature in the furnace before it was taken out. 

In order to compare the properties of Fe-based diamond composites, WC-based diamond composites with and without 1 wt % B were fabricated according to the above procedure. Commercial WC-based matrix powder consisted of 40 wt % WC (99.8% purity, average particle size 115 μm), 10 wt % Ni powder, B powder (including 0 wt % B, 1 wt % B), 50 wt % Cu alloy, and diamond. The designation and composition of samples are shown in Table 1.

The relative density of samples was determined by Archimedes’ method. The hardness was determined by means of a Mod.HRS-150 tester, and the hardness of the Fe-based matrix was characterized by HRB. The hardness of the WC-based matrix was characterized by HRC. The microstructure and compositions were investigated by using scanning electron microscope (SEM, Hitachi S-4800, Tokyo, Japan) with an energy dispersive spectrometer (EDS). Bending strength was measured by the three-point method. Moreover, in order to investigate the wear resistance of different diamond composite samples, the grinding ratio test was conducted. The specimen bending strength was calculated by the following formula:

(1) $$\sigma =3Pl/2bh^{2}$$

where σ is the specimen bending strength, Pa; *P* is the load on the specimen, N; *l* is the length of the specimen bending test holder, 24 mm; and *b* and *h* are the width and height of specimen, 5 mm and 8 mm, respectively.

The wear test conditions were as follows: a 100 mm-diameter and 20 mm-thick SiC grinding wheel was used as a rider; the normal load was 500 g; the liner rotational speed was 15 m/s; grinding time >100 s. The abrasion ratio was calculated by the following formula:

(2) $$R_{a}=\Delta W_{w}/\Delta W_{s}$$

where $R_{a}$ is the grinding wheel-specimen-abrasion-ratio; $\Delta W_{w}$ is the weight loss of SiC grinding wheel, g; $\Delta W_{s}$ is weight loss of sample, g.

## 3. Results and Discussion

**Figure 2:**
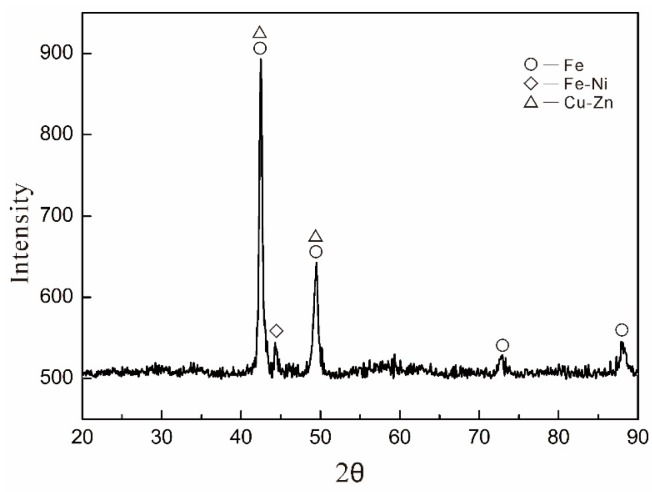
The phase structure of Fe-based diamond composite matrix.

**Figure 3:**
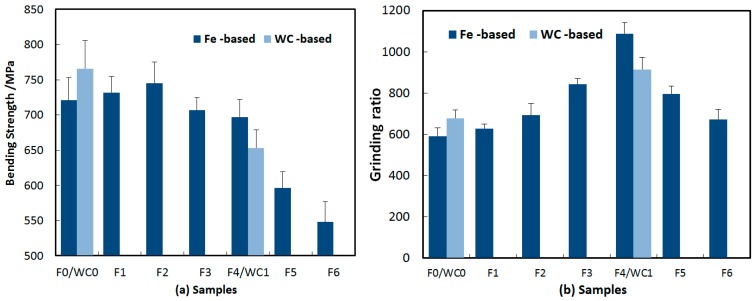
Test results of (**a**) bending strength and (**b**) grinding ratio of samples.

Table 1 shows the relative density and hardness of samples. Fe-based diamond composites have higher relative density than WC-based diamond composites. The hardness of the Fe-based matrix increased with increasing B content. However, the hardness of the WC-based matrix with and without B exhibited little change. The phase structure of the Fe-based diamond composite matrix is shown in Figure 2. There are mainly Fe, Fe-Ni and Cu-Zn XRD patterns included in the figure. Figure 3 shows the test results of sample bending strength and grinding ratios. For sampes WC0 and WC1, bending strength decreased by 15%, and the grinding ratio increased by 35% after adding 1 wt % B. For Fe-based diamond composites with different B content, the bending strength of Fe-based diamond composites decreased with increasing B content, and the grinding ratio initially increased, and then decreased. F4 exhibited an optimal overall performance with the bending strength having decreased by only 3%, while the grinding ratio improved by over 84%. In comparison to WC0 and WC1, it was seen that F0 had a lower bending strength and grinding ratio than that of WC0. However, F4 showed higher bending strength and grinding ratio than that of WC1.

**Figure 4:**
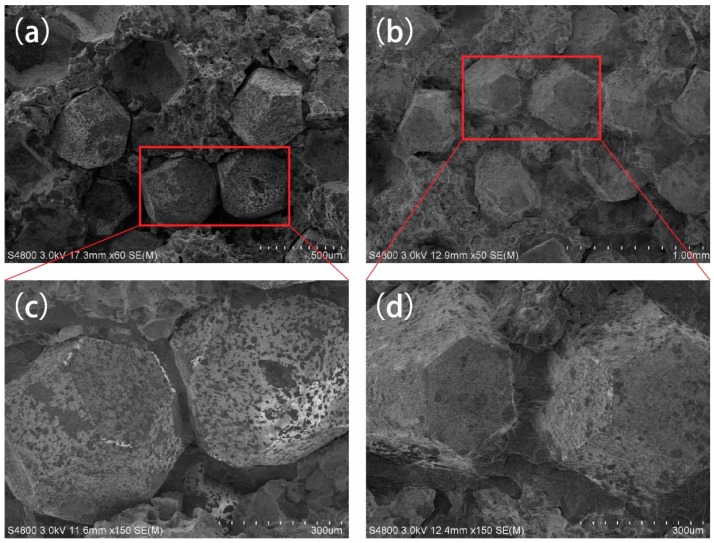
Microstructure of diamond on fracture surface of samples F0 (**a**,**c**) and F4 (**b**,**d**).

Figure 4 shows the microstructure of diamond grits on the fracture surface of Fe-based diamond composites, with and without B. For Fe-based diamond composite without B (Figure 4a,c), it can be seen that the diamond surface is eroded to many etch pits. These etch pits would lower the grade of diamond and reduce its cutting ability [8,13]. For Fe-based diamond composite with 1 wt % B (Figure 4b,d), the diamond grits show integrity in terms of surface and shape edge. The connection between diamond grits and the matrix of F4 was closer than that for F0, under the same bending test. The close connection is of benefit to hold the diamond and prevent it from pulling out in grinding. Apparently, these microstructures have an important influence on the wear resistance of diamond composites.

**Figure 5:**
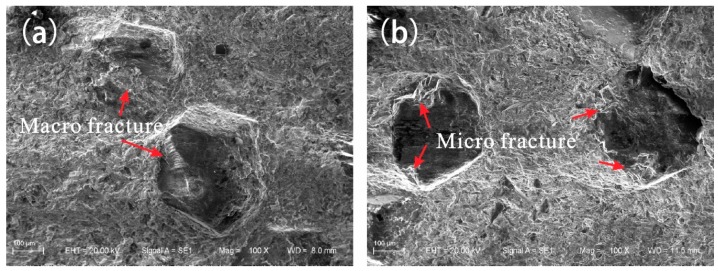
Friction morphology of diamond grits: (**a**) Friction morphology of sample F0; (**b**) Friction morphology of sample F4.

Figure 5 shows the friction surface of diamond composites after conducting the wear-resistance test. The diamond grits of Fe-based diamond composite showed a round macro-fracture shape. The round diamond may lead to bit failure, and the cutting edges of the macro-fracture diamond may significantly affect the cutting ability and lead to loss of its cutting capacity. However, the diamond grits of Fe-based diamond composite with B were mainly present in micro-fracture, increasing the cutting edge of the diamond and improving the cutting ability. In addition, the tail of the diamond grits appeared to be tadpole-shaped, illustrating that the matrix matches the wear of the diamond grits. Thus, F4 had a higher grinding ratio than that of F0.

**Figure 6:**
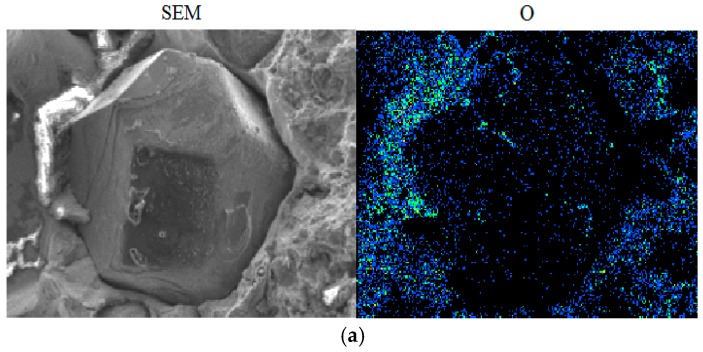

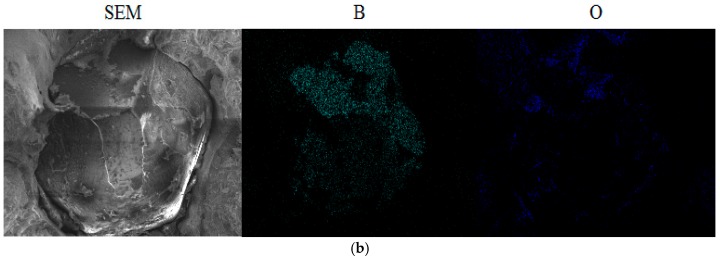
(**a**) O on the diamond surface of Fe-based diamond composites; (**b**) B and O on the diamond surface of Fe-based diamond composites with B.

Figure 6 shows the EDS element distribution mapping of B and O on the diamond surface of Fe-based diamond composites, with and without B. In the Fe-based diamond composite without B, more oxygen appears to be attached on the diamond surface, which is easily oxidized. However, for Fe-based diamond composite with B, B evenly attached on the diamond surface, but less oxygen was on the diamond. Based on the thermodynamic calculation, B more easily reacts with oxygen than carbon. Boron oxide has a good oxidation protection application and wettability on carbon materials below 1000 °C, thus reducing diamond oxidation. In addition, according to Fe-B and B-C phase diagram [23], B can react with Fe and C. Hence, B plays an important bridging role for improving the bonding ability between diamond and matrix by the iron boride and boron carbide. Therefore, B effectively protects diamond from being graphited and oxidized. These reasons lead us to consider the contribution of Fe-based diamond composite with B to wear resistance.

## 4. Conclusions

Fe-based diamond composites, with and without B, were fabricated by pressureless infiltration sintering. The bending strength was decreased, and the grinding ratio was improved with increasing B content. Fe-based diamond composite has an overall optimal performance, with the bending strength decreasing by only 3%, while the grinding ratio improved by over 84%. It is worth noting that B has an obvious effect on the wear resistance of Fe-based diamond composites. In addition, B helps reduce the oxidation of diamond under high temperature.

Fe has proven to be a potential candidate for replacing WC in fabricating diamond bit composites by pressureless infiltration sintering. However, Fe appears to have a serious damaging effect on diamond grits, especially under high temperature; thus, it is necessary to adopt some measures and control the Fe content. This is where B appears to show a good effect on the wear resistance of Fe-based diamond composite; however, the content and underlying mechanisms are worth further study.

## Figures and Tables

**Table 1:** The designation, composition, and physical properties of samples.

Designation	Composition	Relative Density (%)	Rockwell Hardness Scale B (HRB)	Rockwell Hardness Scale C (HRC)
F0	Fe-based Matrix	94	56.4 ± 1.5	–
F1	Fe-based Matrix + 0.25 wt % B	95	58.4 ± 2.1	–
F2	Fe-based Matrix + 0.5 wt % B	97	59.6 ± 1.6	–
F3	Fe-based Matrix + 0.75 wt % B	95	62.4 ± 1.2	–
F4	Fe-based Matrix + 1 wt % B	96	63.5 ± 1.0	–
F5	Fe-based Matrix + 1.25 wt % B	96	65.0 ± 1.4	–
F6	Fe-based Matrix + 1.5 wt % B	95	67.2 ± 1.8	–
WC0	WC-based Matrix	92	–	43 ± 1.0
WC1	WC-based Matrix + 1 wt % B	90	–	44 ± 0.5
